# Borderline personality disorder Athens study: a quasi-experimental pragmatic trial for the assessment of a public, psychodynamic, stepped care service for borderline personality disorder patients

**DOI:** 10.3389/fpsyt.2025.1493265

**Published:** 2025-07-01

**Authors:** Ioannis Malogiannis, Lida Anagnostaki, Maria Aspradaki, Panagiotis Aristotelidis, Katerina Karambela, Maria Amperiadou, Vasiliki Efthymiou, Phaithra Kriezi, Ioanna Theodoridou, Pentagiotissa Stefanatou, George Konstantakopoulos, Kyriakos Souliotis, Lily E. Peppou, Eleni Giannoulis

**Affiliations:** ^1^ Specific Sector of Personality Disorders, First Department of Psychiatry, Eginition Hospital, School of Medicine, National and Kapodistrian University of Athens, Athens, Greece; ^2^ Department of Early Childhood Education, National and Kapodistrian University of Athens, Athens, Greece; ^3^ Second Department of Psychiatry, Attikon Hospital, School of Medicine, National and Kapodistrian University of Athens, Athens, Greece; ^4^ University Research Institute of Maternal and Child Health and Precision Medicine, National and Kapodistrian University of Athens, Athens, Greece; ^5^ Department of Psychiatry, Sismanogleion Hospital, Athens, Greece; ^6^ Department of Speech and Language Therapy, University of Peloponese, Kalamata, Greece; ^7^ First Department of Psychiatry, Eginition Hospital, School of Medicine, National and Kapodistrian University of Athens, Athens, Greece; ^8^ Research Department of Clinical, University College London, Education and Health Psychology, London, United Kingdom; ^9^ Department of Social and Education Policy, University of Peloponnese, Korinthos, Greece; ^10^ Neurosciences and Precision Medicine Research Institute “Costas Stefanis”, National and Kapodistrian University of Athens, Athens, Greece; ^11^ Laboratory of Psychometrics and Neuropsychology, First Department of Psychiatry, Eginition Hospital, School of Medicine, National and Kapodistrian University of Athens, Athens, Greece

**Keywords:** borderline personality disorder, naturalistic design, stepped care, psychodynamic, effectiveness, cost evaluation, health service

## Abstract

**Background:**

Borderline personality disorder (BPD) is a common mental disorder that severely impairs patients’ psychosocial functioning and quality of life and results in prolonged use of health services. Although psychotherapy is recommended as the most effective treatment for patients with BPD, their complex emotional needs can be met in everyday clinical practice by developing integrative, holistic, personalized mental health services tailored to their needs.

**Aim and hypothesis:**

The aim of our study was to evaluate the effectiveness of a specialized psychodynamic stepped-care service for BPD patients. Our hypothesis is that patients receiving this specialized health-care service will show greater improvement in clinical, functional and quality of life than patients receiving a treatment as usual (TAU) service. In addition, specialized health-care services will prove to be more cost effective.

**Method and design:**

A quasi-experimental clinical trial will be conducted. The study is designed to include 212 BPD patients who will be non-randomly assigned to specialized health care services and to two TAU centers. Patients will be recruited at each site following the routine clinical pathways of referral at each center. The primary outcome measures are BPD severity, suicide attempts and hospital admissions. The secondary outcome measures will include measures of general psychopathology, psychosocial functioning, quality of life and retention in treatment. In addition. An economic evaluation from a societal perspective will be conducted.

**Discussion:**

The development of complex individualized stepped-whole care public interventions for BPD patients requires extended research in everyday clinical practice conditions. In this study, we describe the design and implementation of a pragmatic trial to evaluate this type of health service for BPD patients, and we discuss the strengths as well as the problems and how these can be mitigated.

**Trial registration:**

Clinical Trials gov.: ClinicalTrials.gov ID: NCT06392139 (Protocol ID No. 404/06-07-202).

## Background

Borderline personality disorder (BPD) is a common mental disorder characterized by pervasive and persistent instability in interpersonal relationships, identity and affect, and marked impulsivity (1). BPD patients present high rates of comorbid mood, anxiety, substance use, eating, and other personality disorders (2, 3). BPD patients’ quality of life (QOL) is severely impaired across mental and physical dimensions (4, 5), and their social functioning is consistently poor (6). In addition, BPD patients often experience significant emotional crises, engage in self-harm injury, and make suicide threats or attempts (7, 8), resulting in extensive use of treatment services (emergency departments or inpatient hospitals) (9–12). People with BPD commit suicide at a rate of 10% (13). The point prevalence of BPD is estimated to be 1% in community settings (14).

Reports range from 0.7% to 2.7% for lifetime prevalence (15); however, a previous study in a nonclinical sample (16) reported a higher lifetime prevalence of 5.9%. In clinical settings, BPD is one of the most common PDs, with a point prevalence of approximately 12% in outpatients and 22% in inpatient psychiatric populations (14). Furthermore, 6.4% of patients in primary care settings have a BPD diagnosis (10). Epidemiological studies tend to define BPD as a categorical entity. However, compelling evidence suggests that we should understand BPD as a dimensional construct rather than a categorical one (14, 17). Even low levels of BPD symptoms, not only full-blown BPD, are associated with psychiatric comorbidities and functional disability (17).

Previous research has highlighted the increased economic burden of BPD on society due to the extensive use of health services, loss of productivity (11, 18, 19), and intersectoral costs (20). The economic burden of personality disorders, including BPD, appears to be greater than that of depression and generalized anxiety disorder and comparable to that of schizophrenia (11).

Researchers recommend psychotherapy as the first-line treatment for BPD (21, 22). Despite the poor evidence for pharmacotherapy efficacy, clinicians often use psychotropic medications to treat specific symptoms and comorbid psychiatric disorders or when psychotherapy is not accessible (13, 23, 24). Researchers have developed several psychotherapy approaches for the treatment of BPD over the last three decades. It has been shown that cognitive behavioral models (such as schema therapy and dialectical behavior therapy) and psychodynamic therapies (such as transference-focused psychotherapy and mentalization-based therapy) work well and do not cost too much to treat BDP symptoms (e.g., 21, 25, 26).

The increased, complex needs of BPD patients and the need for individualized care for BPD (25), led to the development of different treatment modalities and different integrated programs. These include intensive residential programs (e.g., 28–32), step-down programs (e.g., 33–35), day hospital programs (e.g., 28, 36, 37), and community-based psychotherapeutic models (e.g., 38, 39). These programs are multimodal, may include group and/or individual (mostly psychodynamically oriented) psychotherapy, art and/or drama therapy, psychosocial activities (26, 27) in combination with psychiatric management and symptom-targeted pharmacotherapy (28, 29). Besides, several studies point out the effectiveness of moderate intensity programs compared to more intensive and residential programs ones (26, 27, 30).

In addition, integrated intervention models have been developed that combine cognitive-analytic psychotherapy with general psychiatric care, with a focus on prevention and early intervention for BPD youth (31–33).

However, recent studies still point to a lack of integrative and personalized focus in the treatment of BPD (22, 34, 35). Mental health services for people with personality disorders are still considered to be “doubly disadvantaged”, as they appear to lag significantly behind services for people with other long-term mental health conditions (25). The current literature suggests that people with BPD should receive care that is effective and affordable, that adheres to the general principles of good care for people with personality disorders (36), and that provides rapid post crisis follow-up and prolonged treatment (7, 37). Specifically, there are robust recommendations for integrative whole-service approaches that provide a personalized stepped care model with a combination of psychological and psychiatric treatment interventions, ensuring that patients receive tailored care (7, 22, 25, 38). The involvement of families and relatives in care and therapy is also recommended (34). Although BPD is primarily defined behaviorally and diagnostically, neurobiological research has uncovered consistent disruptions in frontolimbic circuits (39, 40), neurochemical imbalances including serotonergic, dopaminergic, and oxytocinergic dysfunctions (41, 42), and epigenetic mechanisms such as altered gene expression in stress-response systems (43, 44). These findings support integrative models that combine neurodevelopmental vulnerability with environmental stressors to explain emotional dysregulation and interpersonal instability in BPD (45). Despite extensive research into the effectiveness of specific psychotherapies for patients with BPD, there is a lack of pragmatic studies evaluating the effectiveness of stepped health care services that provide treatment for BPD as a whole-care approach (25). Individuals with BPD face both severe psychological symptoms and systemic stigma or neglect. Specialized, integrated treatment programs remain insufficient, particularly within under-resourced public mental health systems. This study advocates for increased investment in long-term, tailored care to address these gaps and promote equitable access to treatment.

Furthermore, in a time of economic crisis, in a budget-constrained health care system, it is crucial to gather information on the costs and benefits of a public sector service to inform decisions on resource investment (46, 47). However, few studies have evaluated the effectiveness and cost-effectiveness of specialized stepped health care services in the public health care system for patients with BPD compared with treatment as usual (TAU). In the 1^st^ Department of Psychiatry of the Medical School of the National and Kapodistrian University of Athens, a psychodynamic specialized stepped health care service named the “Specialized Therapy Program for BPD patients” (STP-BPD) was developed in 1999 (28, 29, 48). Researchers have previously positively evaluated the clinical effectiveness of an earlier inpatient model, which is based on the same psychoanalytic concepts as the STP-BPD (29), but they have not yet investigated the effectiveness and cost-effectiveness of the whole stepped care service in comparison to TAU.

The current service is based on a multimodal treatment approach, offering a variety of specific treatment options, including psychoanalytically oriented group psychotherapy, pharmacotherapy, and monthly follow-up consultations by psychiatrists. All of these treatment services are structured and delivered within a stepped care model, which is consisted of two phases: an initial preparatory one aiming to welcome the patient and provide a facilitating environment to prepare him for the second phase of the specific psychotherapeutic interventions. A second phase where patients are assigned to long term psychoanalytically oriented psychotherapies (mainly group psychoanalytic psychotherapy as well as individual psychoanalytic psychotherapy, group or individual art therapy or a day hospital program. Alongside psychotherapy, patients receive psychiatric management and pharmacotherapy if required. Given the current need for more conclusive verification of the clinical effectiveness and cost-effectiveness of specialized stepped health care services for BPD patients, further research is needed to provide robust evidence on their capacity to produce the aforementioned critical outcomes optimally.

In light of the above, the current article describes and discusses our longitudinal study protocol, which pertains to the research purposes, the implementation context of a specialized stepped care model for BPD patients, our rationale for selecting a naturalistic-pragmatic design, both the primary and secondary outcomes, and the instruments employed for their assessment over a 2-year follow-up. Finally, we discuss the importance of our study aims and the insights they provide.

## Aims and hypotheses

The primary aim of this study was to evaluate whether our specialized stepped care service for BPD patients, namely, the STP-BPD would produce better outcomes for treated patients than would the standard TAU service over a longitudinal 2-year follow-up evaluation at four different time points after participant enrollment. The primary outcomes of this study address the severity of BPD manifestations as well as the symptoms of self-harm and suicidality. Furthermore, we conduct an evaluation of the costs of health care services, as well as an evaluation from a societal perspective. A crucial challenge for BPD-specific health care services would be to improve the general psychopathology; the interpersonal, psychosocial and occupational functions; and the quality of life of patients. These domains are addressed by our study’s secondary outcomes.

Additionally, we will examine the mediating effects of reflective functioning and various aspects related to personality organization, such as defense mechanisms. More specifically, we hypothesize that improvements in reflective functioning during the initial phase of treatment will predict reductions in BPD symptoms and self-injurious behavior at later stages. Age will be treated as a moderator variable in line with evidence regarding the course of the disorder, the symptoms of which, tend to decline with age (49).

In addition to the abovementioned main objectives, this study aims to translate, adapt, and analyze the psychometric properties of the instruments employed in the present research among the Greek population, as some of them have not yet been rigorously evaluated. This investigation is currently in progress for most instruments, apart from those whose Greek versions have already shown satisfactory psychometric properties. Our hypotheses are as follows: 1) Participants receiving specialized stepped care services will exhibit greater clinical improvement, and specialized stepped care services will prove to be more cost-effective than TAU services. 2) Compared with participants receiving standard TAU, those receiving specialized service will exhibit greater functional improvement as well as enhanced QOL. 3) The differences discerned in the outcome variables between the two groups are expected to be mediated by differences in reflective functioning, defense style and personality organization.

Moreover, a longer-term evaluation will take place for both groups but only for the primary outcome variables, as the interview entailing both primary and secondary outcomes is long and by extending the evaluation time frame, we would not want to lose participants at longer follow-ups and risk the emergence of attrition bias.

## Methods and design

### Design

We will conduct our study in a naturalistic–pragmatic design, replicating everyday clinical practice conditions and thereby enhancing its external validity (50, 51). The evidence suggests that well-designed naturalistic studies, compared with RCTs (52–54), can provide evidence without overestimating effect sizes (internal validity), and they can address difficult-to-investigate trial questions, such as those related to public sector services (25). The study is a quasi-experimental pragmatic trial with two groups: an intervention group and a treatment as usual (TAU) group. The treatments for both groups (the intervention group and the TAU group) will be provided at public health hospitals. The intervention group will attend the STP-BPD of the 1^st^ Department of Psychiatry, Medical School, National and Kapodistrian University of Athens, Eginition Hospital. The TAU group will take place in two centers: the Outpatient Clinic for Borderline Personality Disorder Patients of the 2^nd^ Department of Psychiatry Medical School, the Attikon General University Hospital of the School of Medicine (TAU1), and the Outpatient Clinic for Borderline Personality Disorder Patients of the Psychiatric Clinic of the Sismanogleion General Hospital (TAU2). The study established both TAU centers, as detailed below.

**Figure 1:**
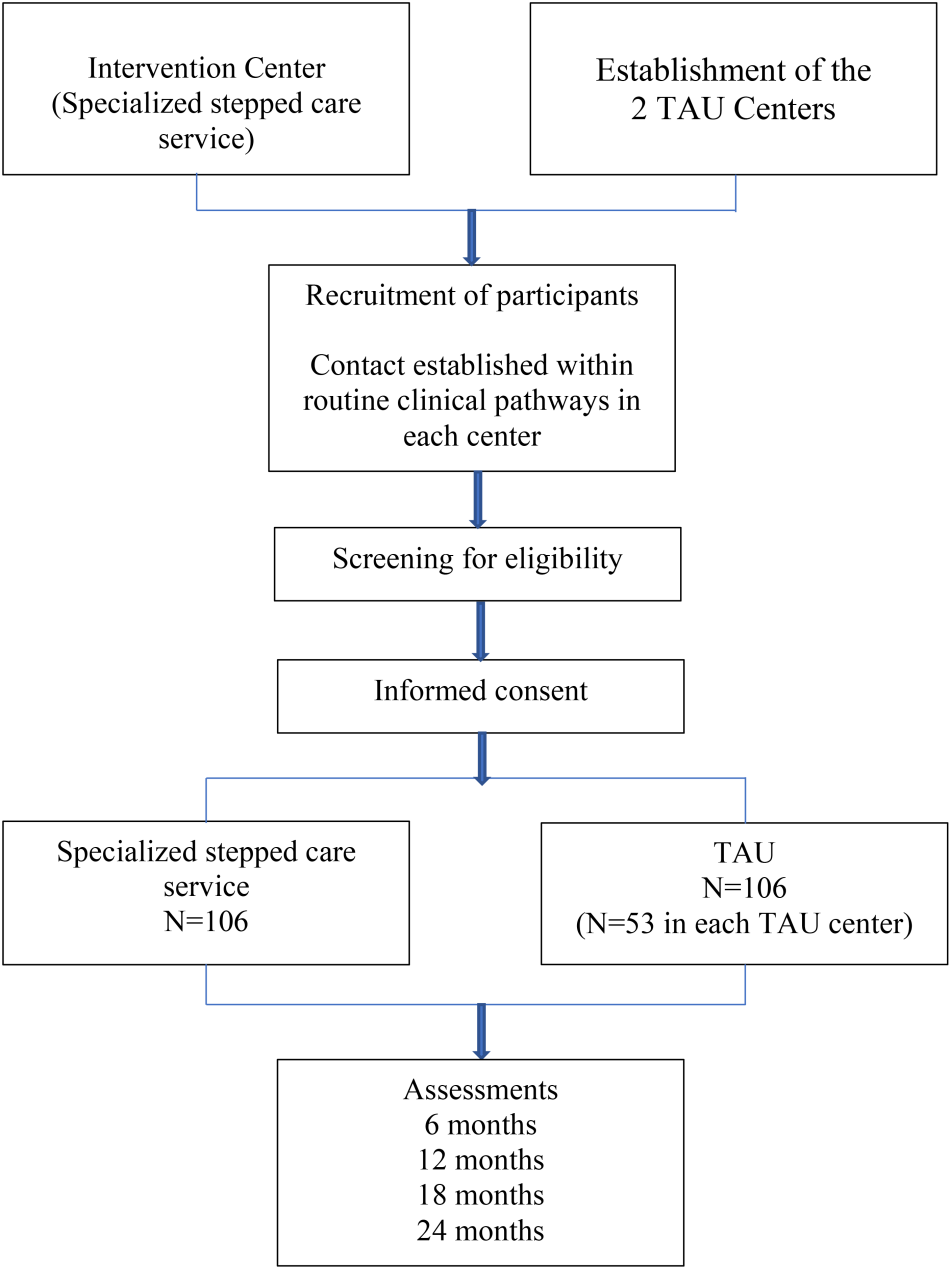
Flowchart of the study design.

We designed this study to evaluate the effectiveness and economic aspects (cost-effectiveness and cost-utility) of the STP-BPD in everyday clinical practice, adhering to the call for *‘real-world’* clinical trials (55, 56). Therefore, we will use minimal exclusion criteria without altering clinical decision-making, health care, or treatment. Patients will be followed for 2 years for primary and secondary outcomes, and for additional 3 years for primary outcomes only. Assessment of the clinical and psychosocial outcome variables will be performed at baseline (T_0_), prior to patient allocation, and every 6 months until the end of the 2^nd^ year of follow-up (T_1_, T_2_, T_3_, and τ_4_). At baseline (T0) and then every year thereafter (τ2 and τ4), the mediators that affect the link between the type of health care service and the major clinical and psychosocial outcomes will be examined. For long-term evaluation, primary outcome variables will also be assessed at T_5_ (2 years and 6 months), T_6_ (3 years), T_7_ (3 years and 6 months), T_8_ (4 years), T_9_ (4 years and 6 months) and T_10_ (5 years). Patient flow, screening and assessments are displayed schematically in **Figure 1**.

Primary outcomes will be (i) the severity of BPD symptoms and (ii) the severity of self-harm and suicidality. Secondary outcomes will be: (i) psychopathological symptom severity, (ii) quality of life, (iii) quality and intensity of interpersonal relations, (iv) functioning and (v) disability.

Mediator variables will be: (i) reflective functioning, (ii) defense style and (iii) personality organization. In other words, better primary and secondary outcomes for the intervention group as opposed to TAU group are expected to be discerned due to differences in reflective functioning, defense style and personality organization. As the design does not include random allocation of participants in the two arms, baseline measurements of the outcome variables will be considered as confounders for the ensuing measurements. Moreover, the two groups will be compared in terms of the following variables: sex, family status, education status, number of children, profession, living arrangement, physical illness, medication for BPD and prior hospitalize physical illness, medication for BPD and prior hospitalization. Those variables with a statistically significant difference between the two groups, will be considered confounder variables in the design and will be taken into consideration as such in ensuing evaluation points. Moreover, information about the treatment delivered in the two groups will be recorded; namely, duration of treatment, type and number of sessions attended. These will be included as confounder variables in the design.

The Ethics Committees of Eginition Hospital, Medical School of the National and Kapodistrian University of Athens (No. 404/05-07-2021), Attikon Hospital (No. 443 25/08/2021), and Sismanogleion General Hospital (27703/10-12-2021) approved the research protocol. The Clinical Trials.gov website has registered the trial under the ID NCT06392139 (Protocol ID No. 404/06-07-202).

### Recruitment

Patients will be recruited from the participating centers. The STP-BPD at the Eginition Hospital receives referrals from public sector psychiatric health services and mental health private practitioners from Athens and the surrounding area. Occasionally, the program receives referrals from other regions of Greece, but for acceptance, a patient must maintain residency in Athens for a minimum of 2 years. The two TAU centers receive referrals from the psychiatric emergency room, inpatient psychiatric unit, or other outpatient clinics of the two hospitals (the Attikon General University Hospital and the Sismanogleion General Hospital, respectively). They also receive referrals from private mental health practitioners. The two TAU centers are located in different areas of Athens, TAU1 in the northern suburbs of Athens and TAU2 in the western suburbs of Athens, thus covering a population from a wide area of Athens and its surroundings with different socioeconomic characteristics. Patients who are already receiving BPD treatment at participating centers will not be included in the study.

All potential participants will be given verbal and written information in detail about the setting, available treatment, assessment procedures, and study purposes. If they agree to participate, they will sign an informed consent form and then be screened for inclusion and exclusion criteria.

### Participants

Patients are eligible if they (i) are between 18 and 55 years of age, (ii) have a primary diagnosis of BPD (diagnosed via the Structural Clinical Interview for the Diagnostic and Statistical Manual of Mental Disorders, 5^th^ version [DSM-5], Personality Disorders; [SCID-5-PD]) (57), and (iii) are willing to participate in the study.

The study will exclude patients with the following conditions (1): lifetime psychotic disorder (excluding a brief psychotic disorder as described in the DSM-5, BPD criterion 9 (1)); (2) bipolar disorder type I; (3) antisocial personality disorder; (4) severe substance dependence leading to severe cognitive limitations during therapy, requiring clinical detoxification; (5) an IQ below 80; (6) organic brain disease; and (7) poor Greek language skills. After referral, following the routine clinical practice of the participating centers, patients will initially be screened for inclusion and exclusion criteria by two experienced psychiatrists in one or two clinical interviews. The SCID-5-PD (57) will then be administered by well-trained, experienced psychologists in separate sessions (see **Table 1**).

**Table 1:** Lists all the measures and their administration dates.

Measure	Screening	T0 (B*)	T1 (6m**)	T2 (1y***)	T3 (18 m)	T4 (2y)	T5 2y6m	T6 3y	T7 3y6m	T8 4y	T9 4y6m	T10 5y
Demographics	✔											
Clinical Interviews (for inclusion & exclusion criteria assessment)	✔											
SCID-5-PD	✔											
BPDsi-IV		✔	✔	✔	✔	✔	✔	✔	✔	✔	✔	✔
Suicide and Self-Harm Inventory		✔	✔	✔	✔	✔	✔	✔	✔	✔	✔	✔
BSI-53		✔	✔	✔	✔	✔						
WHOQOL- BREF		✔	✔	✔	✔	✔						
IIP-64		✔	✔	✔	✔	✔						
WSAS		✔	✔	✔	✔	✔						
WHODAS 2.0		✔	✔	✔	✔	✔						
EuroQol-5D-3L		✔	✔	✔	✔	✔						
Cost Interview		✔	✔	✔	✔	✔						
RFQ		✔		✔		✔						
DSQ-40		✔		✔		✔						
IPO-GR		✔		✔		✔						

*Baseline, **month, ***year

### Sample size

Based on the power analysis (power=0.90), we estimated that the sample size of our study should be 212 participants (106 people per group) to detect differences between the two clinical groups. The power calculation was based on a moderate effect size (Cohen’ s d= 0.45) due to the naturalistic character of the study. A recent meta-analysis (58) established this value of Cohen’s d, calculating the effect size for borderline symptom trials as either 0.31 or 0.56, depending on the stand-alone or add-on design. Accessing these data, the authors decided to set the effect size as 0.45, which is a moderate value that is neither strict nor lenient. Before we reach the aforementioned sample size, we start processing our data within subjects, where a smaller sample size of 158 participants (79 per group) is needed to obtain a significant power of 0.80. The software used to calculate the power was R version 4.0.3.

### Treatments

#### The STP-BPD

In the STP-BPD at Eginition Hospital, people with borderline personality disorder can receive psychoanalytic psychotherapy (59), along with other psychiatric services such as psychiatric evaluation, counseling, medication therapy, and hospitalization if needed (28, 29). In addition to existing treatments, additional therapies (i.e., arts-based therapies) are offered (22). The program bases its integrative approach and clinical practice on Bion’s (60) container/contained model, aiming to establish a broad framework known as the *‘Psychodynamic setting’*. The stable and secure *‘psychodynamic setting’* acts as a receptive container, absorbing the intense projections and projective identifications of the BDP patients, their frequent acting-outs, and the countertransference reactions of the clinicians, and enabling thinking over time, helps the patient internalize the containment capacity (28, 29), which lowers acting-out episodes and improves clinical symptoms.

Central to this approach is its team-based nature, which provides a supportive environment for mental health professionals (61), reduces splitting among them, and enhances their containing function. To this end, weekly staff meetings are held.

As mentioned above, the Program has a stepped care approach with two different steps/phases of intervention:

1. *The reception outpatient clinic* is the initial phase of the intervention, through which BPD patients enter the program. First, psychiatric and psychological eligibility assessments are carried out. Then, for a period of 6–8 months, the reception outpatient clinic offers eligible patients psychoanalytically oriented therapy sessions (1 session per month), psychiatric management sessions, and, if indicated, pharmacotherapy (1 session per month). The first phase of the program, which adheres to a stepped care approach, aims to provide a *‘holding’* environment for patients, manage their ambivalence about therapy, and prepare them for the next phase, which offers intensive psychotherapy. The initial phase also allows for the mental health care of a large number of patients before the more intense interventions of the second phase are available. The capacity of the reception outpatient clinic is 50 patients. Crisis management for patients during phase 1 included visits to the Emergency Department of the Eginition Hospital.

2. *The second phase of the program consists of* sp*ecific interventions*. Clinical criteria (‘readiness for therapy’, self-harm, suicidality, emergency room visits, need for hospitalization, etc.) and availability (e.g., twice a year in therapy groups, once a year in the day hospital, while a small number of patients enroll each year in individual psychoanalytic psychotherapy) determine the assignment of patients to one (or more) of these interventions. The following specific interventions are available:

- *Group psychoanalytic psychotherapy*: This is the main intervention, with a capacity of approximately 50 patients, in groups of 8–10 patients. It lasts for 3 years and offers one or two 90-minute sessions per week.
- *Individual psychoanalytic psychotherapy*: capacity of 4–8 patients. It lasts for 2 years and offers one 45-minute session per week.
- *Group or individual psychodynamic art therapy*: capacity of 4–12 patients. Last 2 years and offers one two-hour group session or one 60-minute individual session.
- *Day hospital program*: The day hospital program can accommodate up to 9 patients. It includes a combination of group and individual psychotherapeutic interventions (psychoanalytic psychotherapy, art therapy, drama therapy) and psychiatric management. The day hospital program lasts 9 months. After their completion, the participants continued in group psychoanalytic psychotherapy for an additional 3 years.
- *Parent groups or parent couples’ professional counseling*: parents of all-day hospital patients and a limited number of outpatients can participate in parent groups or parent couples’ psychoanalytic counseling. It lasts for one year and provides one 90-minute group or couples session per week.
- *Psychiatric management and pharmacotherapy*, if required (1 session per month). Supervision is offered fortnightly for all psychotherapy interventions and weekly for psychiatric interventions.

During phase 2, in case of crisis, the patient contacts his/her psychotherapist or psychiatrist by telephone and, if necessary, goes to the emergency room of Eginition Hospital. In cases of hospitalization, the patient continues the program after discharge.

#### Treatment as usual

TAU includes psychiatric management, counseling and pharmacotherapy (if needed) in the two outpatient clinics for BPD patients (TAU1 and TAU2). The TAU offers one 30-minute session per month. In the event of a crisis, patients contact their psychiatrist during working hours, or after that, they leave a message on the telephone answering machine and, if necessary, go to the Emergency Department of the hospital on duty. As mentioned above, both TAU centers were set up for the purposes of the study to ensure good treatment-as-usual service. They accept only patients with borderline personality disorder, the patients attend regularly (one session per month), and they can contact their doctor directly. These characteristics distinguish TAU centers from other outpatient departments in Greek public psychiatric hospitals, which observe patients across the full spectrum of psychopathology, where psychiatrists are usually not easily accessible, the appointments are made by a telephone operator or a secretary (not by the attending doctor), and sessions are infrequent (usually one session every two or three months).

## Evaluation of clinical effectiveness

### Assessments and clinical outcome measures

Two central research assistants with experience from previous clinical studies received standardized training in the outcome interviews. Training included 3 interview sessions as observers and 2–3 interview sessions under live supervision. When the researchers conducted the interview correctly and the differences in ratings did not exceed 1 point for 3 different items (46), we considered them trained to make independent assessments.

The central research assistants in turn trained a small group of research assistants at each participating center. Throughout the study, the central research assistants guide the research assistants, oversee the scheduling and flow of the assessments, collect and enter data, and ensure the validity of the assessments (62).

The instruments that were not available in the Greek language were translated. Two bilingual speakers (Greek/English) independently translated the instruments into Greek for this purpose, producing a matched version. A professional bilingual translator reverse-translated the agreed-upon version. The back translation was checked for consistency with the original translation. Any inconsistencies were reviewed in consensus meetings and appropriately adjusted. The Greek versions of the above instruments are currently undergoing psychometric property testing. At baseline, the Early Trauma Inventory-Short form (63) will be administered.

### Primary outcome measures

The primary outcome measures include the severity of BPD and severe parasuicidal behavior. 331 The BPDSI (Borderline Personality Disorder Severity Index-Leibniz Institute for Psychology) (64) is a semi structured clinical interview measure that captures specific BPS symptoms over the past three months. It contains 70 items, which are similar to the DSM-IV criteria. It covers nine areas of symptoms: *(1) abandonment; (2) interpersonal relationships; (3) identity; (4) impulsivity; (5) parasuicidal behavior; (6) affective instability; (7) emptiness; (8) outbursts of anger; and (9) dissociation and paranoid thoughts* (64). For its reliability, the internal consistency is greater than alpha = .90 (total score) or between alpha = .49 and.89 (subscales) (65). It is a reliable and valid instrument that is suitable for use as an outcome measure (64, 66). In our sector’s validation procedure of the BPDsi (67, 68), the statistical analyses revealed a fairly valid and reliable tool (Cronbach’s α = .99). A cutoff score of 15 between patients and controls has been established; thus, a score below 15, which is rated two years after randomization or earlier and maintained until follow-up, is used as a criterion for recovery (64).

**Suicide and Self-Harm Inventory** (69): The purpose of the interview is to collect accurate data about attempted suicides and self-harm incidents over a six-month period without attempting to measure their severity. Bateman and Fonagy (70, 71) define parasuicidal behavior as including three types of behavior: *1) suicide attempts (which must be intentional, life-threatening, and require medical intervention); 2) life-threatening self-harm behaviors (which must be intentional, cause visible tissue damage, and require nursing or medical intervention); and 3) admissions to a psychiatric hospital*. The Greek version will be used here (72).

### Secondary outcome measures

**Brief Symptom Inventory (BSI)** (73, 74): The BSI is a 53-item self-report scale that measures psychopathological and psychological symptoms present at the time of assessment. It uses a 5-point Likert scale to rate each item, and the scores cover nine dimensions: somatization, obsession-compulsion, interpersonal sensitivity, depression, anxiety, hostility, phobic anxiety, paranoid ideation, and psychoticism. These nine dimensions can be summed to yield three global indices (73). These synthetic indices are the general severity index (GSI), the positive symptom distress index, and the positive symptom total. Specifically, the BSI uses a 5-point Likert scale ranging from 0 (―not at all to 4 (―extremely). The Cronbach ‘s α for the instrument in total is.96 and ranges between.71 and.87 for its subscales (75).

**The World Health Organization Quality of Life (BREF)** (76, 77): The WHOQOL-BREF is a self-report questionnaire that is short and easy to administer and assesses 4 domains of quality of life (QOL): physical health, psychological health, social relationships, and the environment. In addition, there are 2 items that measure overall QOL and general health. The WHOQOL (Bref) is grouped into 4 domains of QOL and 2 items that measure overall QOL and general health: 1. Physical health, 2. Psychological health, 3. Social relationships 4. Environment. Cronbach’s alpha values for each of the four domain scores ranged from.66 (for domain 3) to.84 (for domain 1), demonstrating good internal consistency (76).

**Inventory of Interpersonal Problems (IIP–64)** (78). The IIP-64 refers to the interpersonal circumplex model (79–81), which has two separate dimensions that describe the quality and intensity of behavior between people. The IIP-64 names its scales *domineering*, *vindictive, cold, socially avoidant, submissive, exploitable, overly nurturing, and intrusive*. Researchers have demonstrated that the psychometric properties of the IIP-64 range from acceptable to good. A study by Vittengl, Clark, and Jarrett (82) on the English version revealed that the subscales were reliable (Cronbach’s alpha) between.72 and.88 and that they were reliable again after 20 sessions of cognitive therapy for depression, with scores of 0.79 for the DOM and 0.84 for the AFF. In our sector’s validation procedure of the IIP-64 (83), the statistical analyses revealed a valid and reliable tool (Cronbach’s α = .932).

**The Work and Social Adjustment Scale** (**WSAS)** (84). It is a self-report instrument that consists of 5 items that are scored on a scale ranging from 0 to 8. We assessed functional impairment in the domains of *work, household, social leisure, private leisure, and family and relationships at the time of assessment.* The reliability, validity and sensitivity to change of the WSAS have been firmly established in samples of patients with different clinical disorders (85, 86).

The World Health Organization Disability Assessment Schedule (WHODAS 2.0) (12, 87) is a standardized assessment tool developed by the World Health Organization that assesses disability-related health conditions in the past 30 days. The measure assesses disability across six domains: cognition, mobility, self-care, getting along with others, life activities, and participation. A profile of functioning is derived across the six domains, as is a general disability score. The WHODAS 2.0 has demonstrated excellent reliability and validity (12). Across the study samples, Cronbach’s α for the overall score of global functional disability was.97, with the domain scale scores ranging from.83 (self-care) to.94 (life activities).

**Treatment Retention:** Dropouts will be recorded. An assistant researcher will contact through telephone any patient who will drop out, and if she/he agrees, an interview will be conducted to elicit the patient’ s perspective about the intervention and the reasons for dropping out. The interviews were semi structured and were developed by the research group of the study.

### Mediators

**Reflective Functioning Questionnaire (RFQ**) (88, 89): This is a brief, screening measure of reflective functioning. It has been developed to assess severe impairments or imbalances in mentalizing, which are typically observed in patients with borderline personality disorder features. Through confirmatory factor analyses, the two factors—certainty (RFQ_C) and uncertainty (RFQ_U)—were found to be fairly different. They remained the same across clinical and nonclinical samples and had good internal consistency and test-retest stability. The RFQ has satisfactory reliability and test–retest reliability. The estimates of internal consistency for RFQ_U and RFQ_C were.77 and.65, respectively, in the clinical sample and.63 and.67, respectively, in the nonclinical sample. The test–retest reliability over a period of 3 weeks was excellent, with *r*_s_ = .84 and.75 for RFQ_U and RFQ_C, respectively, all *p* =0.001 (88).

**Defense Style Questionnaire (DSQ-40)** (90): The 40-item version of the DSQ-40, derived from the original measure developed by Bond, Gardner, Christian, and Siegel (91), is a widely used self-report measure for defense mechanisms because of its easy administration and cost effectiveness. It aims to assess behavior indicative of conscious derivatives of defensive styles that correspond to hypothesized patterns of unconscious psychological mechanisms (90).

The **Inventory of Personality Organization (IPO)** (92, 93) is a self-report instrument intended to measure a patient’s level of personality organization. We constructed the scale to cover five personality dimensions, with the first three serving as the primary clinical scales. These scales assess the degree of identity pathology, the level of immature defensive mechanisms (Primitive Defenses, PD), and the degree of impairment in the ability to test reality (Reality Testing, RT). The three IPO scales demonstrate adequate internal consistency and good test–retest reliability. Lenzenweger (92) reported that the three main dimensions of an IPO’s first psychometric properties in adult and psychiatric samples are internally consistent: identity diffusion (α = .84–.90), reality testing (α = .85–.87), and primitive defenses (α = .80–.87). Confirmatory factor analysis at the item level supported a two-factor structure of the IPO, which is consistent with Kernberg’s model of borderline personality organization (92).

### Process indicators

Since treatment for BPD in both groups (intervention and TAU) is delivered in routine clinical practice, it might be considered as a complex intervention (94). Hence, it is hard to disentangle the effect of the content of the intervention, the intensity and the duration. Information about the number and type of sessions attended will be recorded and manipulated as confounder variables.

### Statistical analyses

#### Assessing the comparability of the two groups

Prior to the initiation of treatment, i.e., at baseline, the two groups will be compared for potential differences in all primary and secondary outcome variables: severity of BPD symptoms, self – harm and suicidality, severity of psychopathological symptoms, quality of life, quality and intensity of interpersonal relationships, functioning and disability. Due to being many outcome variables, MANOVA will be employed. Moreover, the two groups will be compared for potential differences in various socio-demographic variables as well as the presence of physical illness and details of the medication regime for BPD, by employing t-tests for independent samples. In line with this, socio-demographic and other variables that are significantly different between the two groups will be considered to be confounder variables and will be inserted as such in all ensuing analyses.

#### Evaluation of effect

For evaluating the effect of the group assignment (intervention *vs* control) on the primary and secondary outcome variables, multiple linear regression models will be computed for each outcome variables. As predictors the following variables will be inserted: the baseline measurement of the outcome variable of interest, treatment duration, number of sessions attended and all variables that emerged as confounders at baseline (i.e. socio-demographic and other variables that were significantly different across groups).

This analysis will be replicated for all evaluation time points: T_1_ – T_4_. For time points T_6_-T_10_, it will occur only for the two primary outcome variables.

At the end of the study, a Repeated Measures ANOVA with the group assignment (IV) and the 11 time points of assessment (baseline, T_1_ – T_10_) will be performed for the two primary outcome variables.

#### Mediation analysis

Mediation analysis will occur through regression models, path analyses and the Sobel test. For each of the outcomes, three mediation analyses will be performed, with independent variable being the group assignment, In Model 1, the mediator will be reflective functioning, in Model 2, the defense style and in Model 3, the personality organization.

Mediation analysis will occur at T_2_, T_4_, T_6_, T_8_, T_10_ Moderation

In the multiple linear regression models described in the Evaluation of Effect, in the predictor variables, an interaction term between group assignment and age will be inserted as well.

#### Dropout rates

Dropout rates will be compared at the end of the study with the non-parametric criterion Mann Whitney (IV: group assignment and DV: number of drop outs), as number of drop-outs is an ordinal variable.

The Statistical Package for the Social Sciences (SPSS) version 25 (95) will be used for the statistical analyses. All significance tests will be two-tailed, with a 5% significance level and 95% confidence interval.

## Evaluation of cost effectiveness

### Cost measurement

#### Cost interview

An interview will be conducted to retrospectively assess the cost of illness over the past six months. These costs will include direct expenses—such as healthcare-related costs (e.g., hospital visits, psychiatric and psychological consultations, medication use, hospitalizations)—and indirect expenses, encompassing both patient and family burdens (e.g., informal caregiving by relatives or friends, out-of-pocket expenses for substances like alcohol, drugs, and tobacco) as well as broader societal costs, such as productivity loss. The recall period for the interview is 6 months (62).

### Utility measures

A five-item self-report measure (EuroQol-5D-3 L) (96, 97) will be used to assess five different dimensions of patients’ health-related quality of life: self-care, mobility, usual activity, anxiety/depression, and pain/discomfort. Quality adjusted life years (QALYs) based on EuroQol-5D-3 L data will be calculated to compare the quality of life of the participants in the intervention and the TAU programs. The EuroQol-5D-3 L scores range between 0 and 100 to provide a single index measure of the health status of patients.

### Analyses

The economic evaluation will include both a cost-effectiveness analysis (CEA) and a cost-utility analysis (CUA) based on the intention-to-treat principle. The cost interview data will be screened for irrational responses and outliers in patients’ estimates, which will be investigated on a case-by-case basis and, if necessary, dealt with by establishing decision rules. Intermittent missing values are not imputed. The amounts of resource use measured in the cost interview will be multiplied by their corresponding unit costs and then summed to arrive at a total cost. The unit costs will be based on Greece’s average unit prices. As the study covers three years, the unit costs alternate according to the average prices of each year. We also consider productivity losses, informal care, and voluntary work. Using multilevel modeling techniques, the cost-effectiveness and cost–utility ratios will be analyzed.

## Qualitative study on patients’ perspectives

Patient viewpoints are crucial in shaping public policy and offer valuable insights for the enhancement of mental health services (35, 98). Patients randomly selected from the specialized therapy program and from the treatment, as usual, (TAU) centers will be invited to participate in interviews aimed at gathering information regarding their perspectives and experiences with the service and therapy provided. It is projected that interviewing 12 patients in each group will suffice to achieve thematic saturation (99).

## Discussion

This article presents the detailed design of a trial to assess the clinical effectiveness and economic impact of a comprehensive, stepped care service for patients with BPD in the public health sector compared with TAU. Specifically, this quasi-experimental pragmatic trial will assess the clinical, interpersonal, psychosocial, and occupational functioning, as well as the cost-effectiveness and cost-efficiency ratio, of the STP-BPD compared with the TAU. To the best of our knowledge, few studies have investigated specialized whole-care stepped services for BPD. The idea behind this service is that it will have better clinical, functional, and economic outcomes than the TAU by putting resources into psychoanalytically focused, custom-made interventions. The STP-BPD adheres to a stepped, individualized, and integrative approach to the treatment of BPD patients, in line with current literature recommendations (22, 25). On the basis of the container/contained psychoanalytic model (60), the program unfolds in two phases: an initial *‘watchful waiting’* phase (38) and a *subsequent* phase offering various personalized options for long-term psychodynamic interventions. In both phases, psychological and psychiatric treatments are combined to

ensure tailored care on the principle that “not one size fits all” (100). This integrative approach also extends to interventions for carers and support and supervision for staff (61). For the trial, two additional public health units were established to serve as TAU centers. This is particularly important in Greece, where mental health resources for BPD patients are scarce. These TAU centers accept BPD patients exclusively, ensuring regular, monthly consultations and direct communication with their physicians, thus addressing ethical concerns related to patient allocation. A thorough personality disorder assessment is carried out at the time of referral in all participating centers, in line with recent recommendations (61).

The naturalistic design of this study which utilizes minimal exclusion criteria ensuring the participation of patients of everyday clinical practice and which does not alter decision-making regarding the treatment, neither follow treatment manual, guarantees high external validity. While real-world clinical trials may be considered to have lower internal validity because of the nonrandomized allocation of patients, the robust design of this study—including a large, diverse sample, comprehensive outcome measures, and well-defined criteria—also ensures high internal validity. As a result, this study is expected to make a significant contribution to the field of BPD, facilitate healthcare provider training, and promote the development and dissemination of specialized, integrative programs for BPD patients.

Economic evaluation in mental health, particularly for BPD, is challenging owing to the complexity of accounting for all associated costs and outcomes. Despite the lack of consensus on which costs to include, cost-effectiveness analysis remains crucial, especially given the chronic underfunding of mental health services in Greece. Such an analysis will provide valuable insights for the development of specialized programs for BPD patients. This article emphasizes the prevalence and societal burden of the disorder by highlighting the innovative application of the psychodynamic approach in the treatment of BPD, which is now endorsed by the WHO (15). Given the need for conclusive evidence of the clinical and cost-effectiveness of specialized stepped healthcare services for BPD, this study aims to provide robust evidence through a quasi-experimental pragmatic trial. The trial is expected to demonstrate the benefits of psychodynamically oriented, tailored interventions for BPD patients and to contribute to the expertise, training, and development of specialized integrative programs for BPD treatment.

### Expected and potential long-term benefits

For individuals with severe personality disorders, the long-term implementation of comprehensive mental health interventions is expected to significantly improve mental health care and policy. The key benefits include the following:

- Community-Based Support: Enhanced integrated care within communities will improve patient outcomes and foster a supportive environment conducive to recovery and long-term management.
- Destigmatization: Increased awareness and education reduce the stigma associated with personality disorders, encouraging individuals to seek help and promoting the acceptance of mental health issues as vital to public health.
- Rational Medication Use: Programs promote the judicious use of medications, reduce polypharmacy and ensure effective treatments with minimal side effects.
- Reduced Hospitalizations: Effective community-based interventions reduce both voluntary and involuntary hospital admissions.
- Shorter hospital stays: For necessary hospitalizations, the duration of stay is minimized through effective treatment strategies, leading to better outcomes and efficient resource use.
- Fidelity Measures and Monitoring: Developing tools to monitor intervention effectiveness and costs will ensure continuous improvement and accountability.
- Informed Policy Making: Data on mental health service evolution and effectiveness will enable policymakers to create responsive and effective policies.
- Workforce Education: Postgraduate and training programs for treating personality disorders will equip professionals with the latest knowledge and techniques, improving care delivery.

Overall, these interventions promise a more effective and compassionate mental health care system through community support, destigmatization, rational medication use, and data-driven policy making (101).

## Limitations and challenges

Participants could not be randomly allocated to the two arms, as the study was aiming to evaluate the effectiveness of BPD treatment in routine clinical practice conditions (feasibility reasons underlay opting for quasi experimental design rather than an RCT). As a result of this, the comparability of the two groups (intervention *vs* control) is questioned in these designs, especially in terms of selection bias and confounding variables. Patients with BPD from both groups were recruited from services affiliated with hospitals (outpatients departments of hospitals treating patients with BPD), rather than from community mental health centers, in order to keep the severity of BPD at similar levels. Hence, the two services of the control group were selected because they also treat patients with severe forms of BPD and because they are located at different regions of Athens area, with different socio-economic backgrounds. In line with this, it was assumed that the severity of BPD would be comparable across groups as well as their socio-economic background (and pertinent environmental stressors). Differences in baseline measurements between the two groups will be explored in order to confirm or refute the presence of statistically significant differences between the two groups in the outcomes of interest at baseline, that is prior to the initiation of the intervention. Baseline differences will be inserted as a confounder variable in ensuing evaluations (T1 – T10). Moreover, differences between the two groups in terms of cardinal socio-demographic variables and medication regime variables will be explored. Variables that will be substantially different between groups will be considered confounders. Due to timeline constraints, consultation with a statistician is planned for the next stages of the project; this limitation is acknowledged, along with the need to apply more contemporary and robust analytical approaches.

It merits noting that the evaluation of outcomes on many time points (T1 – T10) as well as the direction of causality, intrinsic in longitudinal designs, adds robustness to the study design. Furthermore, the evaluation of a specific pathway of effect, that is that the intervention group will show better scores at the primary and secondary outcomes because of better scores at the mediator variables, strengthens the internal validity of the study. In other words, the study does not only hypothesize that change will be taking place between the two groups in terms of outcome variables but it also clarifies the path whereby change will be elicited (mediation analysis). Inevitably, and due to non-random allocation, the design could not control for unknown confounding variables, which is a limitation of the design. In conclusion, due to the study taking place in routine clinical practice its external validity is high; however, its internal validity is questioned due to the quasi-experimental design. Consistent efforts have been made to increase its internal validity; however, unknown confounding could not have been addressed for due to reasons intrinsic to the quasi-experimental design.

Moreover, patients’ use of additional therapeutic interventions may also be a confounding factor. However, this study only treats patients at the assigned center. In addition, being a prevalent user is classified as an exclusion criterion, which minimizes inception bias (102).

Finally, bias arising from deviations in the care provided from the intended intervention is referred to as performance bias (102). This limitation can be addressed by precisely defining the treatment provided. Nevertheless, no matter how strictly an intervention is defined, there will inevitably be some variation due to possible differences in the experience of care providers. Furthermore, due to treatment for BPD being a complex heath intervention (94) it is hard to disentangle the effects of the content of the intervention, the duration and intensity of treatment. Information on these elements will be gleaned and treated as confounder variables in the model.

Moreover, differences between the two groups in terms of cardinal socio-demographic variables and medication regime variables will be explored. Variables that will be substantially different between groups will be considered confounders. Age will also be explored as a potential moderator variable, based on prior meta-analytic evidence suggesting that younger individuals may benefit less from psychotherapy for BPD (103). Although this finding was based on limited and low-quality evidence from only two adolescent RCTs, it highlights a possible differential effect of age that could extend, cautiously, to young adults. If such an effect is confirmed in our data, it will be taken into account in the outcome analyses.

## Conclusion

The current quasi-experimental, pragmatic trial is designed to investigate how a psychodynamic stepped approach delivered as a holistic, individualized service compares with TAU. In addition to investigating clinical effectiveness, the trial will include an economic evaluation to investigate how the integrative stepped approach compares with TAU. Overall, the trial aims to contribute to an evidence-based understanding that will inform decisions about the optimal forms of care and treatment for patients with BPD, both from a clinical and societal perspective.
